# Consideration of health literacy in patient information: a mixed-methods study of COVID-19 crisis communication in Dutch rheumatology

**DOI:** 10.1186/s41927-022-00283-x

**Published:** 2022-09-07

**Authors:** Mark Matthijs Bakker, Tess Luttikhuis, Polina Putrik, Isabelle Jansen, Jany Rademakers, Maarten de Wit, Annelies Boonen

**Affiliations:** 1 Department of Internal Medicine, Division of Rheumatology, Maastricht UMC, PO Box 5800, 6202 AZ Maastricht, The Netherlands; 2 CAPHRI Care and Public Health Research Institute, Maastricht University, Maastricht, The Netherlands; 3 Nivel Netherlands Institute for Health Services Research, Utrecht, The Netherlands; 4 Tools2Use Patient Association, Amsterdam, The Netherlands

**Keywords:** Health literacy, Crisis communication, Patient information, COVID-19

## Abstract

**Background:**

The rapid spread of COVID-19 required swift action to provide people with rheumatic and musculoskeletal diseases (RMDs) with reliable information. People with limited health literacy constitute a vulnerable group that might have difficulty accessing, understanding and applying health information, particularly in times of crisis.

**Objectives:**

This study explored (a) key aspects of crisis communication and (b) explicit consideration of people’s health literacy needs in communication to people with RMDs during the first wave of COVID-19 in the Netherlands.

**Methods:**

We conducted a convergent, qualitatively driven mixed-methods study comprising seven qualitative interviews with professional representatives of organisations responsible for information provision to people with RMDs, and quantitative analysis of 15 patient information materials distributed by these organisations. The study was guided by principles of crisis communication and health literacy. We assessed understandability and actionability of information materials using the Dutch version of the Patient Education Materials Assessment Tool (PEMAT, resulting in a percentage of quality criteria met), and language difficulty level using an online application (assessing difficult words, jargon, passive, complex and long sentences, long paragraphs, and difficulty levels according to the Common European Framework of Reference for Languages (CEFR, from A1 (basic) to C2 (proficient))).

**Results:**

Respondents reported lack of preparedness, challenges related to scientific uncertainty and reaching the target group, difficulty simplifying information, and uncertainty regarding adequacy of the communication approach. Patient information materials (written and video) showed variation in actionability (range 60–100%) and understandability (range 58–100%), and 69% of written materials were too difficult, mostly due to the use of long sentences and difficult words. The quantitative findings were in coherence with the limitations in communication reported by respondents. Several potential improvements were formulated in ‘lessons learned’.

**Conclusions:**

Although rheumatology organisations mostly adhered to principles of crisis communication and made efforts to adapt information to their audience’s needs, we propose recommendations to improve preparedness, strategy, content, reach and consideration of health literacy needs in future crisis communication.

**Supplementary Information:**

The online version contains supplementary material available at 10.1186/s41927-022-00283-x.

## Key messages

- Rheumatology organisations reported efforts to align with principles of crisis communication and health literacy.
- Patients’ health literacy needs were insufficiently considered and patient information materials were too difficult.
- Improving preparedness, collaboration between organisations and disciplines, and adaptation to patients’ needs is necessary.

**Supplementary Information:**

The online version contains supplementary material available at 10.1186/s41927-022-00283-x.

## Introduction

The rapid global spread of Coronavirus Disease 2019 (COVID-19) raised acute concern among the general population [1], especially among people with pre-existing conditions that possibly made them more vulnerable to infection or prone to a severe course of COVID-19 [2, 3]. Lee and You [4] observed higher levels of perceived susceptibility and perceived severity of disease among people with lower health status. Particular groups of concern include people with pre-existing respiratory problems [5], people undergoing chemotherapy [6], and people with inflammatory rheumatic and musculoskeletal diseases (RMDs) treated with immunosuppressive drugs, including people with Rheumatoid Arthritis (RA), Spondyloarthritis (SpA), and systemic diseases [7, 8]. At the Maastricht University Medical Center + (Maastricht UMC +), the first phone calls and emails with questions and concerns from patients reached the outpatient clinic on February 27^th^, 2020, the day of the first confirmed case of COVID-19 in the Netherlands. Immediate action by healthcare providers and organisations was required to provide reliable, timely information to people with RMDs. Important issues included the risk of infection and severe COVID-19 in subgroups of patients, continuation of non-steroidal anti-inflammatory drugs (NSAIDs) and specific antirheumatic drugs such as disease modifying antirheumatic drugs (DMARDs) or glucocorticoids, safety at work, temporary closure of clinics and organisation of (semi-) virtual care, and the alleged role of antirheumatic drugs in the treatment of COVID-19.

The World Health Organization (WHO) and Centers for Disease Control and Prevention (CDC) set out principles to consider in crisis communication at the time of an outbreak [9, 10]. Among others, these documents provide governments and organisations with advice on being prepared, being credible, and achieving reach and impact through a communication strategy that fits the needs of the targeted audience [9, 10].

The needs of the audience can be diverse and depend on, for example, people’s clinical profile or socioeconomic background. People with limited health literacy constitute a vulnerable group that might have specific health information needs, particularly in times of crisis [11–13]. Health literacy is defined as “the combination of personal competencies and situational resources needed for people to access, understand, appraise and use information and services to make decisions about health. It includes the capacity to communicate, assert and act upon these decisions” [14]. Adequate health literacy is of vital importance to be able to navigate the abundance of health information of differing quality, deal with scientific uncertainty, and adequately assess risks and adapt health behaviour accordingly [11–13]. In realisation that limited health literacy is prevalent both in the general Dutch population [15] and among patients with rheumatic diseases [16], consideration of health literacy principles in crisis communication is required. In this paper, we therefore explored (a) key aspects of crisis communication and (b) the explicit consideration of people’s health literacy needs in communication with people with RMDs during the first wave of COVID-19 in the Netherlands.

## Methods

We conducted a convergent, qualitatively driven mixed-methods study [17, 18] in which we interviewed professional representatives of organisations with different roles in providing information to persons with RMDs in the Netherlands during the COVID-19 pandemic. The qualitative findings, distilled from a generic qualitative approach [19], are combined with a quantitative assessment of patient information materials provided by these organisations in the first months of the pandemic, to further understand how principles of crisis communication and health literacy were applied in communication to persons with RMDs. We used a separative approach, in which qualitative data and quantitative data analyses are conducted independently before integrating the datasets for further interpretation [18].

### Sampling of organisations

We purposefully sampled four organisations: two large patient organisations, the national association of rheumatology professionals, and the rheumatology department of one academic medical centre to gain insight into crisis communication on a national and hospital level. While the primary audience differed between organisations, communications by all four organisations were used to inform patients with RMDs with diverse health literacy needs, and therefore fit the scope of this study. Each organisation was asked to refer us to one or several professional spokesperson(s) on this topic. All respondents were involved in one-on-one or mass communication with patients, and/or responsible for coordinating COVID-19 communication, and could therefore reflect upon the process as ‘expert insider’.

### Qualitative data collection—interviews

In May and June 2020, two researchers (TL & IJ) conducted semi-structured interviews via video- or telephone call using an interview guide (Additional file 1). Respondents were asked to describe the crisis communication of their organisation in light of key principles of crisis communication [9, 10] and identify points for improvement. Moreover, we enquired about whether explicit efforts were made to adapt crisis communication to populations with health literacy needs.

### Qualitative data processing and analysis—interviews

Upon the respondent’s consent, the interviews were recorded and transcribed. In one case, extensive notes were taken instead, as close to verbatim as possible. Two researchers (TL & IJ) independently performed open line-by-line coding [20], using Atlas.ti software. We initially developed a coding tree based on the interview guide (deductive coding [20]), but expanded and adapted it after every interview, with emerging codes added (inductive coding [20]). After agreeing on the final coding tree with a third researcher (MB), all interviews were recoded where necessary. In case of disagreement between the researchers, differences were discussed and resolved. Through further axial coding [20], a thematic structure emerged [21]. This thematic structure led us to create a framework comprising four core pillars of crisis communication, i.e. (1) *preparedness,* (2) *strategy,* (3) *reach,* and (4) *content of communication* (Box 1), which we used to describe and structure the results.

**Box 1:** Core pillars and underlying principles of crisis communication and health literate communication [9, 10, 22]

**Pillar 1: preparedness**	
*Planning & guidelines:* A crisis communication plan, developed in non-crisis time, should be readily available [9]	
**Pillar 2: strategy**	
*Announcing early:* Accurate, comprehensive, transparent information should be shared early to build trust and facilitate behavioural change [9, 10] *Frequency:* Information should be updated regularly, to reinforce earlier messages and keep the attention as a credible source [10] *Consistency:* Messages across organisations should be consistent, as people inform themselves using different sources [10] *Transparency:* Information should include acknowledgement of uncertainty, what is known and (still) unknown, and what actions are being undertaken to deal with the crisis, to maintain public trust and promote more deliberate decision-making [9, 10]	
**Pillar 3: reach**	
*Trust:* Communication with the public depends on building, maintaining and/or restoring trust as a precondition for medical advice to be believed and followed, and to ensure it truly reaches the intended audience. Trust is built through long-term relations with the public, acknowledging people’s struggles as well as, counterintuitively, scientific uncertainty [9, 10] *Accessibility (1/2):* Information should be accessible through multiple channels (besides oral communication in clinical encounters) to enhance reach and impact, as a diverse audience is best reached in diverse ways [10]	
**Pillar 4: content of communication**	
*Understanding the public:* Crisis communication should be a dialogue, where providers communicate a message that is adapted towards the needs of their intended audience. Messages should address the concerns that live among the population [9, 10] *Actionability:* Communication should include information on what the public can do themselves (for example in prevention, treatment, or coping). Materials are actionable when consumers of diverse backgrounds and varying levels of health literacy can identify what they can do based on the information presented [9, 10, 22] *Accessibility (2/2):* Information should be accessible in terms of understandability and difficulty, to promote the audience’s understanding [10, 22]	
**Recurring theme: health literacy**	
Health literacy needs of the audience should be considered throughout. This specifically refers to actionability and understandability, but also considers people’s health literacy needs across all principles.For example, this includes using appropriate channels, building trust, and providing tailored guidance and support as a strategy to make sure the communication is understood and acted upon by the audience	

Note: Accessibility refers to different aspects, some related to reach, others to content

The framework was inspired by key publications [9, 10, 22], and encompasses known principles of crisis communication and health literacy that help contextualise and understand our results. While using this framework enhances the interpretability of our findings, note that there is some dependency and overlap of principles between and across core pillars, indicating that crisis communication is more complex than the pillars in this framework seem to suggest. Consideration of health literacy throughout the process was described and evaluated separately, because the analysis suggested it was a recurring theme across the other themes, rather than a separate pillar. Whenever relevant, we distinguish between mass communication and one-on-one communication in describing the results.

### Quantitative data collection—patient information materials

To complement the qualitative findings and gain insight in the outputs of the crisis communication efforts described by the respondents, we conducted a quantitative assessment of patient information materials used by the four organisations, in parallel with the qualitative data collection and analyses. We identified patient information materials (texts and videos) provided on websites and social media (Twitter and Facebook) pages of the organisations between February 27th and June 1st 2020 for assessment. Materials were selected if they a) aimed specifically at an RMD patient audience, and (b) provided information or health advice related to COVID-19. We further included a standardised written communication used at the hospital to support nurses in answering patients’ questions by telephone and individual emails.

### Quantitative data processing and analysis—patient information materials

**Fig. 1:**
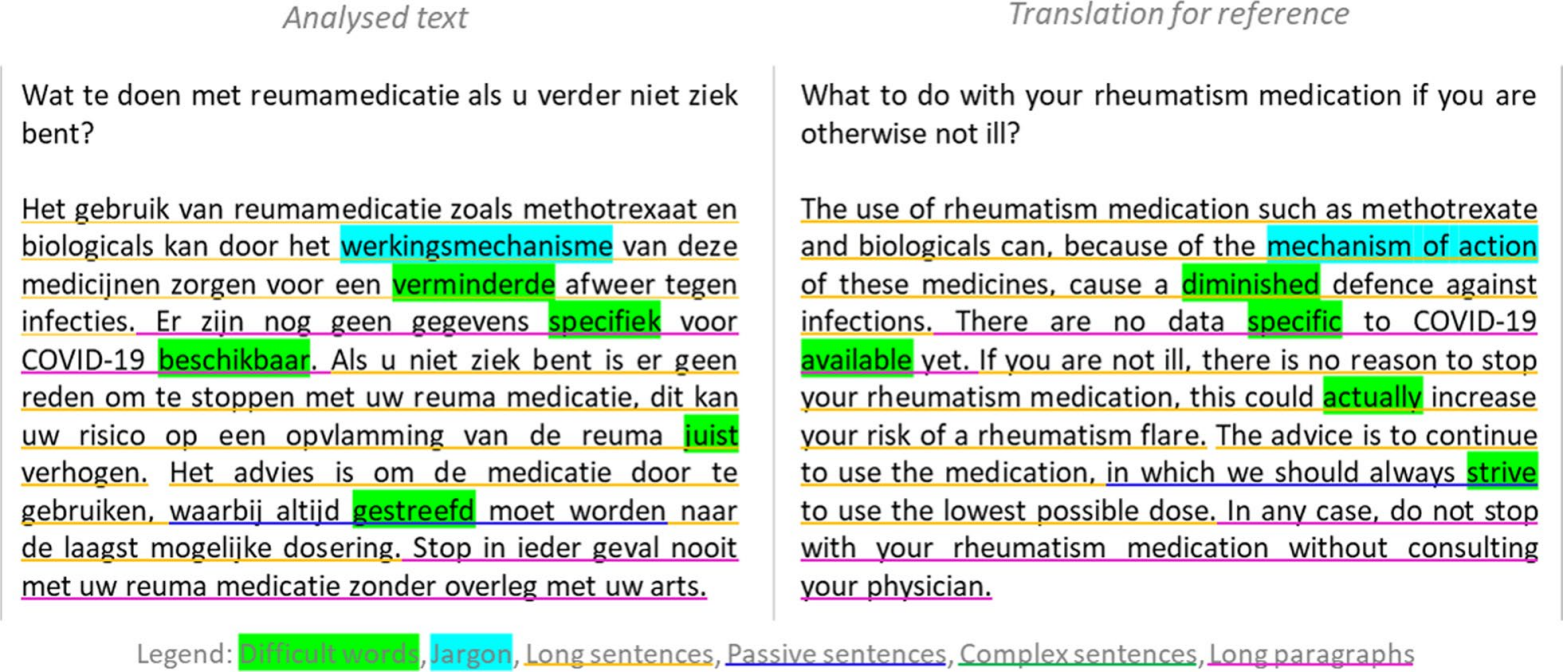
Example of textual assessment using the *‘Klinkende Taal’* [SonaLing] application. Note: Dutch-language text was used for analysis; the English translation is provided as a reference only and may not be equivalent in difficulty level

Written materials were assessed for *difficulty level* using ‘*Klinkende Taal’* [English-language version: SonaLing] [23]. This online application assesses the use of difficult words, jargon, passive, complex and long sentences, and long paragraphs, and assigns difficulty levels according to the Common European Framework of Reference for Languages (CEFR, from A1 (basic) to C2 (proficient)). There is broad consensus that difficulty should not exceed B1 level (lowest level of independent proficiency, indicative of adequate literacy) for the majority of the population to be able to read and understand everything that is written [24–27]. Figure 1 displays an excerpt of an assessment for the reader’s insight.

Both video and written materials were assessed for *actionability* and *understandability* using the ‘*Voorlichtingsmateriaal BeoordelingsInstrument’ (VBI)* [Translated and cross-culturally adapted version of the English-language Patient Education Materials Assessment Tool (PEMAT)] [22, 28]. *Actionability* refers to the extent to which the audience would be able to identify a specific course of action*. Understandability* comprises difficulty of words and sentences, but also factors such as layout, clarity of what concept is discussed, distracting content, and use of illustrations. *VBI* is an easy-to-use freely available Dutch-language checklist in two versions: one for written materials and one for audio-visual materials, comprising 24 (17 for understandability and 7 for actionability) and 17 (13 for understandability and 4 for actionability) criteria, respectively. Two assessors (TL, IJ) separately judged for each applicable criterion whether or not it was met. Discrepancies were discussed and resolved. For each material, the proportion of (applicable) criteria met is given as a percentage for actionability and understandability separately. Hence, a higher percentage score indicates a more understandable or actionable information material. The number of applicable criteria may differ between materials and thus is not comparable across materials.

### Data integration

**Fig. 2:**
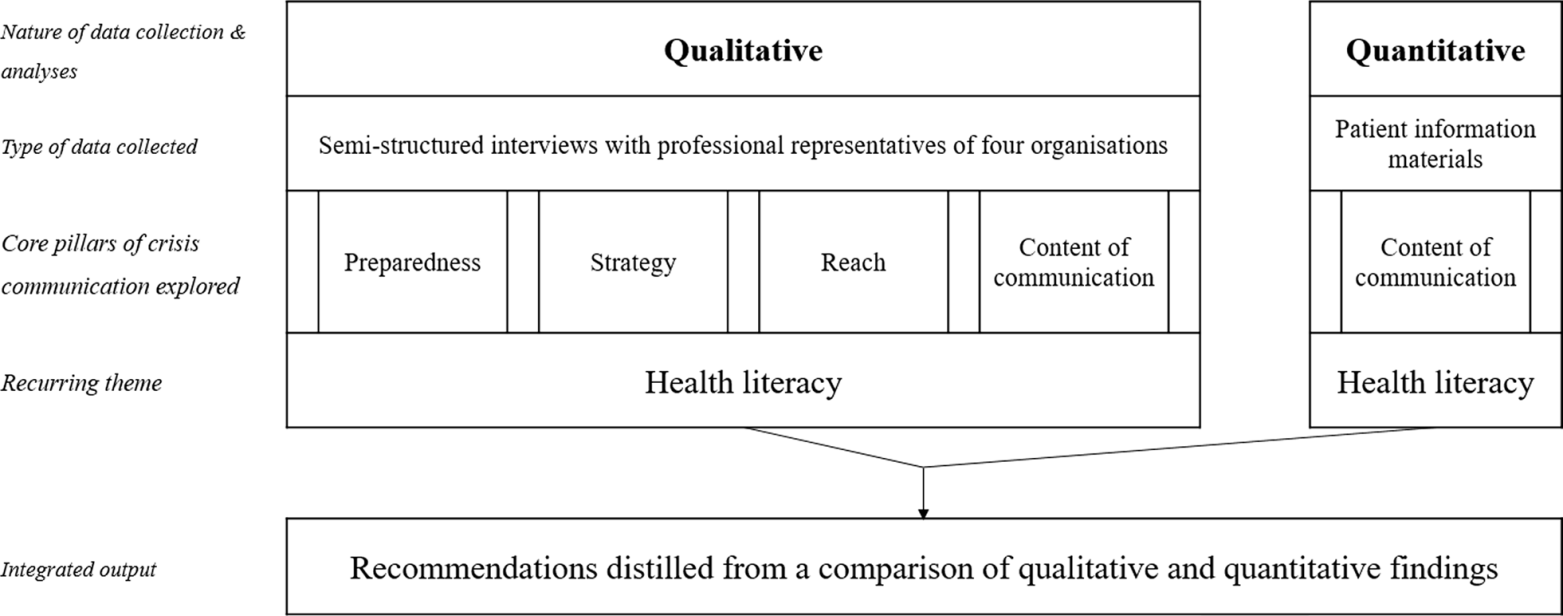
Overview of study design and methods used in relation to the pillars of the crisis communication framework

The convergent, qualitatively driven study design and methods used in relation to the pillars of the framework are displayed in Fig. 2 [17, 18]. While the qualitative data describe the perspectives of professionals on the crisis communication efforts of their organisation in light of key principles, the quantitative data were used to assess the outputs of these efforts, specifically with regards to actionability, understandability and language difficulty of the patient information materials delivered by the organisations (‘content of communication' pillar and recurring theme of health literacy). We merged our qualitative and quantitative datasets at the stage of data analysis [17], to enable mixed-methods analysis and comparison of the ‘content of communication’ pillar and the recurring theme of health literacy. The mixed-methods research question answered in this paper is: to what extent are the outcomes of quantitative assessment of patient information materials in coherence with the perceptions of professionals working for the organisations who produced these materials? [17, 29] We used a contiguous ‘integrating through narrative’ approach, in which the qualitative findings are described first, followed by the quantitative findings [29]. Lessons learned, summarized in the discussion as recommendations, are distilled from a combination of both types of data.

### Ethics and quality considerations

No ethical approval was sought for this study given it involved interviews with professionals in the field and analyses of public information. Respondents provided informed consent before participation and their anonymity was ensured. Researchers conducting the interviews (TL, IJ) and analyses (MB, TL, IJ) were not involved in the crisis communication, and worked independently from the respondents. The corresponding author (MB) was acquainted with five of the respondents prior to the study. Interviewees were asked to review the manuscript to check the interpretation of their statements, which did not result in significant changes.

## Results

All persons invited accepted the invitation to be interviewed. Interviews lasted between 30 min and 1 h. Seven interviews were conducted, one with a spokesperson of patient communication from each of the three invited national organisations, two with rheumatologists responsible for clinical care and overall management of the department of rheumatology of the academic medical centre, and two with rheumatology nurses from the same department who were in direct contact with patients.

### Preparedness

All respondents revealed their organisation had no pre-existing plans, *guidelines* or previous training on the concept of and skills required for crisis communication, and confirmed that this contributed to chaos in the early stages.

“There was absolutely no preparation on what to share with patients or not, what objective, credible information is. It was complete chaos, to be honest. (…). We did not have a crisis protocol available telling us what to do when there’s an outbreak or something heavy happens. Nothing.” (Respondent 1)

Respondents indicated that having a plan with clearly defined responsibilities assigned to specific individuals, and advice on what and how to communicate with patients at times of crisis would be of benefit. In addition, *crisis communication training* should be offered to those responsible for patient communication.

“Imagine a new crisis occurs, then you should have a protocol ready, to have your information provision run smoothly from the beginning. (…). You can never predict exactly what a crisis situation is going to be like, but with the experience of the COVID-19 pandemic, we can make a more general crisis protocol.” (Respondent 5)

### Strategy

All organisations immediately started screening the available information from scientific sources, as well as documenting incoming questions from patients and professionals, to provide patients with answers to frequently asked questions in a timely fashion.

“We established that information rather quickly, and those frequently asked questions were updated as time passed, for example when schools partially reopened we added some specific information.” (Respondent 4, referring to mass communication)

Nevertheless, only one organisation provided information *early*, i.e. on the day of the first confirmed case in the Netherlands, while the other organisations took over a week for the first public announcements.

All organisations strived to update patients *frequently* regarding new developments. However, respondents from the hospital revealed that technical and time constraints at the organisational level hindered regular updating of disease-specific information on the website.

The organisations made efforts to ensure *consistency* of information across organisations. Although the association of rheumatology professionals answered questions of individuals that contacted them directly, they did not seek to inform patients themselves. Instead, they *collaborated* closely with one patient organisation and the hospitals by providing information that was agreed upon by a COVID-19 working group, which in turn ensured *consistency* with communications by the Dutch government and EULAR COVID-19 working group. Information was then disseminated to a wide patient audience by the patient organisation and healthcare providers. One patient organisation did not collaborate explicitly with other organisations, but contributed to *consistency* by adapting information from other Dutch and European resources for their audience. Respondents suggested coordination between partners could be improved by: (1) appointing a single point of contact in each organisation and governmental agencies (to be included in a crisis protocol), (2) sharing developed audio-visual materials between organisations, and (3) further increasing collaboration on a European level.

“We should have that. If something happens, which person is the one to talk to? Then you can immediately get together. Direct communication. We already had that, we already had direct communication with the professionals’ association, but I still think it is good to appoint one person.” (Respondent 6, referring to mass communication)

Several respondents emphasized that in mass communication, they remained *transparent* with their audience about scientific uncertainty and that scientific developments followed each other rapidly. In most cases, they explicitly referenced where information came from and specified that information was based on the most recent insights, implying these might change over time. In clinical encounters at the hospital, however, information was sometimes personalised and presented in a more certain, directive manner, to avoid further confusion or unnecessary anxiety. This relied on the treating professional’s judgement of whether the patient would benefit from strong recommendations as opposed to transparency and acknowledgement of uncertainty.

“I try to read my patients, and well, some people can or cannot handle uncertainty, they can or cannot assess the impact of uncertainty. Of course, I can misread my patients, but I do try to add nuance. And if I feel like this person is not going to understand, I did it [provide information] in a more direct way.” (Respondent 7, referring to one-on-one communication)

### Reach

Organisations used *multiple channels* to share information. Healthcare professionals provided information one-on-one during (usually remote) consultations, by telephone, or email, and all organisations used their websites. However, interviewees mentioned this was not sufficient, as it relies on patients to actively search for the information. The patient organisations used digital newsletters to reach their members and their social media pages (Twitter, Facebook and Instagram) to reach a wider audience. One organisation hosted a video livestream combined with questions & answers (Q&A) with a physician.

Organisations indicated they likely benefitted from established *trust* of their audience. The national organisations often communicated directly with their formal members, and healthcare providers acknowledged the importance of trust and the personal relationship with their patients to ensure adherence to health advice, despite *scientific uncertainty*.

“We explained time and time again that while the medication perhaps could negatively impact their risk, the risk of a flare of their rheumatic disease would be more dangerous, because you might need a lot more medication [immunosuppressive medication to control a flare]. And those are definitely bad for you.” (Respondent 7, referring to one-on-one communication)

At the same time, respondents noted that they also communicated with patients that were new to them, because they were new to the clinic or normally under regular care of a colleague, making it more difficult to use a personalised *trust-based approach*.

“Most patients who contacted me knew me, and I knew them. That creates confidence. It is nice to be in contact with people you know, so you can give personalised advice. But there are also people who had recently been diagnosed who are not familiar with everything yet, these people are more difficult to reassure. That is much more difficult.” (Respondent 5, referring to one-on-one communication)

Respondents indicated that the reach of information could have been better. For example, healthcare professionals wished *tailored* letters had been sent out to specific patient groups to increase reach, also allowing for more specification and nuance, for example about using specific medication. Other respondents wondered how to reach people who might not seek out information themselves.

“And yeah, then it is good to realise, like oh, if you want to reach two million people [= total potential audience of interest in the Netherlands], that you will have to use more social media. That you have a plan for that. A social media plan. A press list. That you can work through very different channels (…) outside the rheumatology channels. (…) I think such a list of networks and contacts, that you can use that to distribute information in such occasions. Actually, always, I have to say. Actually we could do a lot better in daily practice as well, when it comes to distribution of news.” (Respondent 1, referring to mass communication).

### Content of communication

The respondents agreed that they *prioritised* repeating the key messages of risk reduction (hygiene, physical distancing and avoiding people with symptoms) and continuation of rheumatic medication. Furthermore, they tried to adapt information to the *needs of the audience*, taking into account both the patients’ and professionals’ perspective on these needs. However, only limited initiative was undertaken to actively uncover information needs from a patient perspective. One organisation actively monitored social media to uncover *patient information needs*; other respondents stated they found out about patients’ needs through their questions, making adaptation primarily a reactive process. Moreover, there was no system in place to find out if the population deemed the information provision to be adequate, with the exception of feedback during the Q&A session, which clearly filled a need.

“What really helped was the Q&A session with a rheumatologist. (…) All those questions about medication and corona were asked. And since that session, we typed out all frequently asked questions. Everything we encountered, all those questions, yeah, that really took the pressure off.” (Respondent 6, referring to mass communication)

Especially in the earliest stages, respondents noticed that information materials were *difficult to understand.* Materials were heavily text-based, as visual materials took longer to develop.

“We worked on a (…), a general poster to share on social media. With icons for hand hygiene, sneeze in your elbow, continue using your medication. We worked on that, but by the time that was completely done, with the right pictures and everything, it was a month and a half later.” (Respondent 1, referring to mass communication)

One organisation explicitly said they attempted to send out information at a B1 *difficulty* level only. Other respondents said that they tried to use simple language, but acknowledged it may have been too difficult for some patients.

“The emails, definitely, that was just plain text, (…) quite an extensive piece of plain text. I think it was communicated like that on the website too, which is indeed unfortunate.” (Respondent 3, referring to mass communication)

Respondents suggested further identification of and adaptation to the needs of patients would be necessary in future crises, for example by considering patients with *multi-morbidity*. Collaboration with experts from other medical specialties is required to achieve this. Another specific suggestion was the issue of dealing with *fake news* about medication often used by patients with rheumatic diseases.

“When it came to ace-inhibitors, chloroquine, or anti-inflammatories, there was quite a bit of fake news about those at some point. And for some patient groups, specifically our patients, (…) we could have specifically targeted this group, actively informed them…“ (Respondent 3, referring to mass communication).

### Health literacy

All interviewees acknowledged that this crisis was particularly difficult for people with limited health literacy. There were substantial amounts of information to process and many of the guidelines were difficult to understand.

“We did not manage to do that.” [about adapting to health literacy needs] “We were happy to even be able to share information at all. (…). But the information is so incredibly complicated, no matter how hard you’ve worked on a clear message. You notice, especially when explaining medical information, that you lose people” (Respondent 1)

Furthermore, the majority of information was shared online, while patients with lower (e-)health literacy might struggle to use digital services. As there were no crisis communication plans in place, health literacy needs were not explicitly taken into account from the onset. Notwithstanding, all four organisations had already initiated efforts to address health literacy needs before the pandemic, mainly with regard to understandability of materials, and continued these during the pandemic. On that line, respondents adopted a one-size-fits-all approach by aiming to use easier vocabulary in conversations with patients, writing patient information at B1-level, and experimenting with social media and interactive webinars. One patient organisation consulted patient language ambassadors and health equity experts to review their website and some information materials. Nevertheless, these intentions did not always result in accessible information for people with limited health literacy, due to *time-pressure*.

“I do think people with limited health literacy get the short end of the stick. (…). We were not able to, considering the pace at which information was delivered and the pace at which we had to make decisions and share information in our own words, I think it happened at the expense of readability.” (Respondent 1, referring to mass communication)

Respondents at the hospital tried to adapt information to the health literacy needs of individual patients by speaking in clear language, keeping instructions as simple as possible and focusing only on the most important issues. Two respondents noted they strongly preferred telephone calls to emails, as it allowed them to check if their message had come across. A challenge, however, was to *judge* the patient’s health literacy struggles.

“If you know your patients, it is easier than if you have a patient you do not know that well. That is a lot more difficult to estimate, like, have they really understood or are they just afraid to tell me that they have not understood.” (Respondent 2, referring to one-on-one communication).

One respondent noted the importance of using as many channels as possible, to increase reach and allow people to ask questions in a way they prefer. Another respondent suggested that in the future, patients should be able to opt for instructions at their preferred difficulty level. Further collaboration between organisations as well as training or guidelines on how to reach people with health literacy needs were recommended. Importantly, one respondent reminded us always to include the *patient perspective*.

“So to always keep the critical view of the patient involved. Keep an eye on what’s going on here, what’s going on there? (…). And don’t think that you already know! That happens a lot in healthcare, certainly also in rheumatology, where the specialists are real people’s doctors, who might feel like they know what patients think. But it really is different if you’re a patient yourself. (Respondent 6, referring to health literate communication).

### Quantitative assessment of patient information materials

Thirteen texts and two videos (between two and five per organisation) were analysed (Table 1 and Additional file 2). Assessment of *difficulty* of texts revealed that only four out of thirteen texts (31%) shared with patients were written at the aspired B1-level, despite several respondents indicating they aimed to write at this level. Admittedly, the professionals’ association wrote their two texts primarily for a professional audience, but these texts were also provided to patients. Most problematic across different texts was the use of difficult words and long sentences. The use of long paragraphs was only a problem in materials of one organisation.

**Table 1:** Summary of assessment of patient information materials per organisation

	VBI [PEMAT]		Klinkende Taal [SonaLing] Online application
	Actionability	Understandability	Difficulty level
Hospital (n = 4)	80–100%	67–89%	B1–C1
Patient organisation A (n = 4)	100%	67–100%	B2*
Patient organisation B (n = 5)	60–100%	58–87%	B1–C1
Professionals’ association (n = 2)	80%	75–89%	B2

Displayed results represent the range of scores (difficulty levels or percentages, depending on the tool applied) for all materials per organisation. Percentages indicate the proportion of applicable quality criteria for actionability and understandability that were met. The number of applicable criteria may differ between materials. VBI = Voorlichtingsmateriaal BeoordelingsInstrument [Patient Education Materials Assessment Tool (PEMAT)]. Further details in Additional file 2. *Only written information materials (n = 2)

Assessment of *actionability* revealed that while some materials allowed the audience to clearly identify a specific course of action, others lacked a direct appeal for action or clear steps to take. Median actionability of the assessed materials was 80% (range 60%—100%). Lower actionability was observed in more general materials about dealing with fake news, and the effects and availability of rheumatic medication, provided by a patient organisation.

The texts with lowest *understandability* were those that also scored poorly in terms of language difficulty. Least understandable were the text provided to patients by email and on the website of the hospital, and the texts about fake news and medication. Median understandability of texts was 83% (range 58%—100%). Both videos scored 67%. While many materials were highly understandable (8 materials scored between 80–100%), almost all left room for improvement. In addition to using easier language, texts would benefit from a better use of images, visual cues such as bullet points or bold text to highlight importance, and removal of distracting information. Both videos lacked a clear thematic structure and a summary.

The quantitative analyses supported the respondents’ qualitative reflections that efforts to provide understandable information were made. At the same time, these analyses also confirmed the limitations in communication acknowledged by the respondents in the qualitative interviews.

## Discussion

This study explored key aspects of crisis communication and the explicit consideration of people’s health literacy needs in communication to people with RMDs during the first wave of COVID-19 in the Netherlands. Furthermore, it explored whether professionals’ perceptions on the quality of patient information materials were in coherence with quantitative assessment. In summary, the patient organisations, the professionals’ association and the academic hospital reported explicit efforts to provide people with RMDs with relevant, timely and accurate information through multiple channels. While these efforts generally aligned with principles of good crisis communication, the respondents acknowledged several limitations such as a lack of preparedness, lack of reach to specific groups, lack of insight into patients’ needs, and high difficulty and low understandability of patient information materials. The quantitative assessment confirmed that the majority of texts was too difficult to understand, and often lacked actionability. Therefore, we propose several recommendations for future crises, especially in informing patients with diverse health literacy needs (Box 2). We deem these lessons learned transferable across countries and medical specialities.

**Box 2:** Recommendations for improvement of crisis communication

**Preparedness** *(qualitative)*	
Use the experience of this pandemic to establish a future crisis communication plan, by reviewing and amending the ad hoc protocols that were established	
Train staff and management in crisis communication and health literate communication	
**Strategy** *(qualitative)*	
Build sustainable relationships with relevant organisations to ensure consistency in messages	
Inform people early and frequently, preferably in a way tailored to clinical profiles	
Remain transparent about uncertainty	
**Reach** *(qualitative)*	
Use multiple channels to communicate your messages, including those that do not rely on the patient’s initiative (active outreach)	
Use different outreach strategies to cater to a diverse audience, also beyond the clinic’s regular patients and the associations’ own members	
**Content of communication** *(qualitative* + *quantitative)*	
Adapt information to different people’s needs, considering e.g. age, cultural background. Actively discover these needs from the patient perspective *(qualitative)*	
Ask your audience for suggestions and feedback and use it to revise your strategy and provided information *(qualitative)*	
Combat fake news through acknowledgement and counterarguments *(qualitative)*	
Check difficulty level of written information (aim at A2/B1) and adapt accordingly *(quantitative* + *qualitative)*	
Make sure information is directly applicable in practice *(quantitative* + *qualitative)*	
**Health literacy** *(quantitative* + *qualitative)*	
Explicitly consider people’s health literacy needs throughout and provide tailored guidance and support, beyond merely simplifying written health information	

In brackets it is indicated what source data the recommendation was based on (qualitative or mixed-methods)

We argue for the consideration of broader aspects of health literacy needs in a crisis communication approach, beyond readability of information only. While we asked respondents whether health literacy of patients was explicitly considered, implying a broad definition, most reflections focused specifically on difficulty and understandability of information. However, simplifying texts to a B1-level is insufficient for a proportion of the population with a lower reading level (people with low literacy or illiteracy, estimated at 14%) [24, 25, 27]. Moreover, health literacy needs manifest in diverse ways in practice, with patients exhibiting different strengths and weaknesses across domains of health literacy, thus understanding information might not be the main problem [16]. None of the organisations reported to have accounted for this diversity in health literacy needs by considering tailored guidance and support. This is unfortunate because besides a general risk of patients being underinformed and underprepared [11–13], recent research further emphasizes the importance of considering health literacy needs in crisis communication. While several studies reported that people with lower health status [4] and people with rheumatic diseases [30] were aware of their vulnerability and therefore took precautions [30], this may not have been true for people with limited health literacy within those groups, who were found to perceive themselves less susceptible to COVID-19 infection [31, 32] and were possibly less likely to take preventive measures [33]. A Dutch qualitative study on the COVID-19-related challenges of people with a chronic illness and limited health literacy highlighted the important role of one-on-one communication by trusted healthcare professionals, especially in providing information tailored to the health literacy needs and clinical profile of the individual [34]. Knowing that COVID-19 has exacerbated health inequalities [35–37] and health literacy plays a role in vaccine hesitancy [38, 39], the need to consider health literacy in crisis communication is imperative.

Admittedly, the principles of crisis communication as suggested by the WHO and CDC [9, 10] implicitly overlap with principles of health literate communication. Communication with the public should in both cases be timely, simple, coherent and consistent, and provided messages should be understandable, actionable and adjusted to the audience’s needs, which implies consideration of health literacy diversity. Nevertheless, this study shows that consideration of the specific needs of people with limited health literacy was delayed and limited to a basic definition, rather than explicitly and consistently taken into account from the start. Knottnerus, Heijmans and Rademakers [34] showed that this was not unique to the rheumatology context, but extends across people with chronic diseases in the Netherlands. While the intersection between crisis communication and health literacy should be further explored, investing in training and guidelines for health literate communication for organisations and health professionals will potentially be useful in general patient communication as well as in times of crisis.

This paper contributes to the fast-increasing body of scientific literature about the role of health literacy in COVID-19 communication. Levin-Zamir et al. [40] described multiple case studies showing the need to focus on health literacy at multiple levels of the social-ecological model (individual, interpersonal, organisational, community and policy level) in order to be more prepared for future crises, and prevent problems such as care avoidance, mental health issues, or lack of adherence to public health guidelines. Ratzan, Sommariva and Rauh [41] offer lessons learned in global health communication early in the pandemic, summarized as “be proactive”, “plan ahead” and “focus on people”. In the age of social media, this means considering not only people’s health literacy needs, but also their media literacy needs to help people appraise content and consequently make sound health decisions [41]. Along this line, Hamaguchi, Nematollahi and Minter [42] argue for the use of visual aids to leverage the power of social media and reach a wide audience with simple, accessible health information. The recommendations proposed in the present paper reinforce those made by these scholars.

The findings in this study should be seen in light of a few limitations. Firstly, this study explores the application of key principles of crisis communication and health literacy based on views of a limited number of respondents, and does not provide a comprehensive assessment of the national information provision in rheumatology. Moreover, the focus of our study was the Dutch context, and we only assessed Dutch-language materials. Our findings may therefore not be directly transferable to non-Dutch speakers in the Netherlands. Nevertheless, we sampled diverse actors of importance in the Dutch context to get a broad idea of the quality of the initial response to the COVID-19 pandemic while minimizing the burden for respondents and organisations, and have uncovered general lessons learned to inform future improvements. Because these lessons are generally not context-specific, they may also be of inspiration to other countries and medical specialties to critically reflect on their crisis communication. Secondly, we cannot be sure of the full reach and impact of the strategies employed by the organisations, because we did not interview patients. We decided against patient interviews for feasibility and desirability reasons, as to not further burden patients at a very high-stress period in time. Reflections of difficulty are therefore based on the expert respondents’ observations, supported by quantitative evidence. Future research should nevertheless focus on the patient perspective to complement our findings and recommendations, as was also recommended by a respondent. As a promising starter, while there was no mention of the reach and impact of communication, Décary et al. [43] draw attention to the patient perspective throughout and beyond the pandemic, particularly in considering most vulnerable patients in the implementation of new care models, and investigation into the uncertainties that people with RMDs face in different aspects of their lives. In addition, the REUMAVID study, a cross-sectional online survey conducted among patients with RMDs in several European countries (not including the Netherlands), showed that 45.6% of surveyed patients had not received rheumatology-specific information at all [44]. Patient associations were reported as the most frequent source of information [44]. While this study did not assess actionability, understandability and difficulty of information and communication from the patient perspective, the results are indeed indicative of overall room for improvement in crisis communication.

## Conclusions

In conclusion, despite being underprepared for a crisis communication campaign, the patient organisations, professionals’ association and an academic hospital demonstrated aspects of good crisis communication with some consideration of health literacy. Analyses of their experience resulted in several lessons learned for future crises, to improve crisis communication in general, but particularly to consider patients’ health literacy needs.

## Supplementary Information

**Additional file 1**. English translation of interview guide. Text document with the interview guide used in for the qualitative interviews. The guide was translated to English and provided as additional material for the readers’ insight.

**Additional file 2**. Details of patient information materials assessment. Further details of the patient information materials assessment are provided as additional material. Results are shown as range of scores of patient information materials assessment per organisation, and specified per material.

## Data Availability

The datasets generated and analysed during the current study are not publicly available due to the respondents not consenting to the raw data being shared publicly, but are available from the corresponding author on reasonable request.

## Abbreviations

CDC: Centers for Disease Control and Prevention
CEFR: Common European Framework of Reference for Languages
COVID-19: Coronavirus Disease 2019
EULAR: European League Against Rheumatism
PEMAT: Patient Education Materials Assessment Tool
Q&A: Question & Answer
RA: Rheumatoid Arthritis
RMDs: : Rheumatic and musculoskeletal diseases
SpA: Spondyloarthritis
VBI: Voorlichtingsmateriaal BeoordelingsInstrument (Dutch abbreviation)
WHO: World Health Organization
