# Supplementary material for: Consideration of health literacy in patient information: a mixed-methods study of COVID-19 crisis communication in Dutch rheumatology
Source: BMC Rheumatol. 2022 Sep 7;6:52. doi: 10.1186/s41927-022-00283-x (PMC9449268; doi:10.1186/s41927-022-00283-x)
Supplement: Supplementary file 1 — Additional file 1. English translation of interview guide. Text document with the interview guide used in for the qualitative interviews. The guide was translated to English and provided as additional material for the readers’ insight. [file 41927_2022_283_MOESM1_ESM.docx]

**Additional material 1: English translation of interview guide**

1. **How were you prepared for providing information about COVID-19?**
   1. Were there any guidelines for crisis communication which you could rely on?
   2. Did you previously receive any training about crisis communication?
   3. Where did you find the information that would be shared with patients?

- Overall, did you feel well prepared to provide information about the Corona crisis? Can you imagine any improvements?

1. **How was the provision of information about COVID-19 to patients organised in your division or organization?**
   1. Were there any differences in how information was provided during the crisis compared to the normal communication of your organization?
   2. How were the tasks divided? Was there anyone specifically responsible for updating patients?
   3. Was there any guarantee that all patients were reached with the information? / was there a strategy to reach as many patients as possible?
   4. When did you start sharing information, how frequently and how was this determined?
   5. Was there any communication with other organizations (such as ReumaNederland, NVR, MUMC+, hospitals, ReumaZorgNederland)?
      1. How did you try to ensure information from these different organizations was consistent?

- Overall, how would you evaluate the organization of the information provision? Can you imagine any improvements?

1. **What was the content of the information which was provided to patients?**
   1. What was the most important information to share to patients for you?
      1. Were there any specific instructions you provided to patients?
      2. Were there any suggestions for patients to improve their own situation?
   2. How did you deal with the uncertainty surrounding the information about COVID-19?
      1. Did you provide any information about the source of information or any decisions made?
      2. Why did you or did you not choose to share information that was still uncertain?
   3. What platforms did you use and what types of information did you provide?
      1. Did you take the difficulty of language into account when providing this information? Did you use any explanatory images?

- How would you evaluate the information provision and guidance for patients who might struggle to understand healthcare information? Can you imagine any improvements?

1. **How did you experience the contact with patients?**
   1. To what extent did you know the patients you contacted?
   2. Did you receive any questions from patients and were you prepared to answer them?
   3. Did you use any specific strategies to help patients understand the information?
   4. Was there a way for patients to provide any feedback on past information or on information they would like to receive?

- Were there any difficulties in the contact with patients? Can you imagine any improvements?

1. **Closing questions:**
2. If there would be some other crisis where crucial information should be shared with patients, what would you like to see different from how you currently shared information and what you like to keep the same?
3. According to you, what could this crisis teach us about information provision to all patients?
4. Are there any other aspects you would like to discuss?
