# Supplementary material for: Consideration of health literacy in patient information: a mixed-methods study of COVID-19 crisis communication in Dutch rheumatology
Source: BMC Rheumatol. 2022 Sep 7;6:52. doi: 10.1186/s41927-022-00283-x (PMC9449268; doi:10.1186/s41927-022-00283-x)
Supplement: Supplementary file 2 — Additional file 2. Details of patient information materials assessment. Further details of the patient information materials assessment are provided as additional material. Results are shown as range of scores of patient information materials assessment per organisation, and specified per material. [file 41927_2022_283_MOESM2_ESM.docx]

**Additional material 2: Details of patient information materials assessment**

|  | **VBI [PEMAT]** | | **Klinkende Taal [SonaLing] online application** | | | | | |
| --- | --- | --- | --- | --- | --- | --- | --- | --- |
|  | Actionability | Understandability | Difficult words | Passive sentences | Difficult sentences | Long sentences | Long paragraphs | Measured level |
| **Hospital** | **80 – 100%** | **67 – 89%** | **2 – 17%** | **0 – 14%** | **0 – 11%** | **0 – 26%** | **0%** | **B1 – C1** |
| Text 1 | 100% (2 n/a) | 89% (8 n/a) | 2.3% | 0% | 4.3% | 21.7% | 0% | B1 |
| Text 2 | 100% (2 n/a) | 83% (5 n/a) | 7.5% | 0% | 0% | 0% | 0% | B1 |
| Text 3 | 80% (2 n/a) | 67% (5 n/a) | 16.7% | 14.3% | 7.1% | 21.4% | 0% | B2 |
| Text 4 | 80% (2 n/a) | 67% (8 n/a) | 10.5% | 10.5% | 10.5% | 26.3% | 0% | C1 |
| **Patient Organisation A** | **100%** | **67 – 100%** | **5%** | **7 – 12%** | **4 – 6%** | **20 – 23%** | **0%** | **B2** |
| Text 1 | 100% (1 n/a) | 92% (5 n/a) | 4.5% | 7.4% | 5.8% | 19.6% | 0% | B2 |
| Text 2 | 100% (2 n/a) | 100% (8 n/a) | 4.9% | 11.5% | 3.8% | 23.1% | 0% | B2 |
| Webinar 1 | 100% (1 n/a) | 67% (4 n/a) | - | - | - | - | - | - |
| Webinar 2 | 100% (1 n/a) | 67% (4 n/a) | - | - | - | - | - | - |
| **Patient Organisation B** | **60 – 100%** | **58 – 87%** | **7 – 12%** | **2 – 30%** | **2 – 20%** | **9 – 42%** | **0 – 33%** | **B1 – C1** |
| Text 1 | 80% (2 n/a) | 80% (2 n/a) | 6.8% | 7.4% | 1.8% | 9.3% | 0% | B1 |
| Text 2 | 80% (2 n/a) | 87% (2 n/a) | 7.7% | 1.6% | 1.6% | 14.3% | 33.3% | B1 |
| Text 3 | 60% (2 n/a) | 60% (2 n/a) | 11.5% | 29.6% | 18.5% | 33.3% | 14.3% | C1 |
| Text 4 | 100% (2 n/a) | 83% (5 n/a) | 9.4% | 11.5% | 11.5% | 34.6% | 0% | B2 |
| Text 5 | 60% (2 n/a) | 58% (5 n/a) | 8.8% | 9.7% | 19.3% | 41.9% | 14.3% | C1 |
| **Professionals’ association** | **80%** | **75 – 89%** | **7 – 8%** | **0 – 2%** | **4 – 9%** | **8 – 37%** | **0%** | **B2** |
| Text 1 | 80% (2 n/a) | 89% (8 n/a) | 7.6% | 0% | 3.8% | 7.7% | 0% | B2 |
| Text 2 | 80% (2 n/a) | 75% (5 n/a) | 6.8% | 2.3% | 9.3% | 37.2% | 0% | B2 |

**Legend:** Displayed results represent the range of scores (difficulty levels or percentages, depending on the tool applied) of patient information materials assessed per organisation, and specified per material. Percentages for *VBI* assessment represent the proportion of applicable actionability / understandability criteria met. n/a represents the number of statements which were not applicable to the material. Percentages for the *Klinkende Taal* online application indicate the proportion of words, sentences or paragraphs deemed more difficult than at B1-level. VBI = Voorlichtingsmateriaal BeoordelingsInstrument [Patient Education Materials Assessment Tool (PEMAT)].
